# Heterologous Overexpression of *Magnaporthe oryzae* Effector *PWL2* Enhances Rice Blast Resistance via SA-Mediated and *PWL2*-Derived siRNA Defense

**DOI:** 10.3390/plants14213312

**Published:** 2025-10-30

**Authors:** Xiaoqian Sun, Qijing Fu, Lixia Wu, Yu Yang, Hao Luo, Qian Dong, Saijie Li, Yiting Zhao, Xuan Zhou, Suqin Xiao, Jinlu Li, Zaiquan Cheng, Sheng Peng, Qiaofang Zhong, Yunlong Du

**Affiliations:** 1 College of Plant Protection, Yunnan Agricultural University, Kunming 650201, China; 18690435114@163.com (X.S.); qijing_fu@163.com (Q.F.); wlx17584546080@163.com (L.W.); haohluo@163.com (H.L.); 15187885261@163.com (Q.D.); 15198854592@163.com (S.L.); zyt369963@126.com (Y.Z.); zhouxuan@xtbg.ac.cn (X.Z.); mollab@sina.com (S.P.); 2 Hebei Key Laboratory of Horticultural Germplasm Excavation and Innovative Utilization, Hebei Higher Institute Application Technology Research and Development Center of Horticultural Plant Biological Breeding, College of Horticultural Science & Technology, Hebei Normal University of Science & Technology, Qinhuangdao 066004, China; 3 Yunnan Key Laboratory of Cell Metabolism and Diseases, Center for Life Science and School of Life Sciences, Yunnan University, Kunming 650500, China; yangyu110218@163.com; 4 Institute of Biotechnology and Germplasm Resources, Yunnan Academy of Agricultural Sciences, Yunnan Provincial Key Lab of Agricultural Biotechnology, Key Lab of Southwestern Crop Gene Resources and Germplasm Innovation, Ministry of Agriculture and Rural Affairs, Kunming 650205, China; xiaosuqin227@126.com (S.X.); li_jinlu@yeah.net (J.L.); czquan-99@163.com (Z.C.)

**Keywords:** rice, *Magnaporthe oryzae*, effector, *PWL2*, salicylic acid, small RNA

## Abstract

Fungal effectors play an important role in plant immunity. The *Magnaporthe oryzae* effector PWL2 plays a significant role in rice blast disease caused by this fungus. However, the function of PWL2 in rice immunity is not fully understood. In this study, transgenic rice lines overexpressing *PWL2* showed resistance to rice blast. Subcellular localization showed that PWL2-GFP fusion protein is localized on the plasma membrane and cytoplasm. Salicylic acid (SA) induces rice resistance to *M. oryzae*. Notably, the expression of the *NPR1* gene exhibited a rhythmic pattern during the early stages of *M. oryzae* infection in the transgenic rice lines. However, during later stages of infection, transgenic plants showed reduced levels of the *NPR1*, *WRKY45*, *PR1a* and *PR10a* genes, along with decreased H_2_O_2_ accumulation, while SA levels remained unchanged. Transcriptome analysis revealed that SA treatment induced the expression of the *ARGONAUTE11 (AGO11)* gene in rice. Furthermore, during the later infection stage in the transgenic rice lines, the expression levels of both *AGO11* and *PWL2* genes increased. Intriguingly, *PWL2*-derived small interfering RNAs (siRNA) were detected in these transgenic rice lines. It suggests that both the SA signaling pathway and *PWL2*-derived siRNAs function in rice resistance to blast disease caused by *M. oryzae*.

## 1. Introduction

The effector of fungus *Magnaporthe oryzae* plays a critical role in causing rice blast disease. Rice exhibits two main innate immune responses against *M. oryzae* infection: pathogen-associated molecular pattern (PAMP)-triggered immunity (PTI) and effector-triggered immunity (ETI) [1,2]. Fungal effectors can translocate across the plasma membrane (PM) into rice cytoplasm [3] and suppress host immunity to promote fungal growth [4]. The effector pathogenicity toward weeping lovegrass 2 (PWL2) is a host-specific avirulence (Avr) protein [5,6] belonging to the *PWL* family [7,8], and it can translocate via clathrin-mediated endocytosis (CME) into rice cytoplasm and move into uninvaded neighbor cells [9,10]. Sakuranetin enhances rice resistance to blast disease by inhibiting the endocytosis of PWL2 [11]. Whereas, PWL2 is shown as a virulence factor that suppresses host immunity in transgenic barley lines overexpressing *PWL2* [12], and its expression is activated by an unknown signal commonly present in living plant cells [13]. The function of *PWL2* in rice blast disease resistance following *M. oryzae* infection has remained partially unclear.

Salicylic acid (SA) is an important phytohormone in plant defense and is involved in rice resistance to rice blast [14,15,16,17]. The transcription factor WRKY45, which acts in the SA signaling pathway, is also involved in resistance to rice blast [18,19,20]. Small RNAs are involved in rice blast resistance [21,22,23,24], and host-induced gene silencing (HIGS) transgenic rice plants showed significant resistance against *M. oryzae* [25]. ARGONAUTE (AGO) proteins are a core component of the RNA-induced silencing complex (RISC) essential for siRNA biogenesis in plants [26,27]. *Arabidopsis ago4-2* mutant is not affected in SA perception [28]. SA can induce the production of small RNAs that contribute to antiviral immunity [29,30]; however, whether it also induces the generation of siRNAs derived from the *M. oryzae* effector to confer rice blast resistance remains unclear.

In this study, transgenic rice lines overexpressing *PWL2* showed resistance to rice blast. The SA signaling pathway contributes to blast resistance in transgenic rice during the early stages of *M. oryzae* infection. During later infection stages, transgenic plants generated *PWL2*-derived siRNAs but did not exhibit SA-induced resistance. These results demonstrate that the SA signaling pathway and *PWL2*-derived siRNAs function at distinct stages of rice resistance against blast disease. This study provides novel insight into the mechanistic collaboration between SA and siRNAs derived from effector genes in enhancing plant disease resistance.

## 2. Results

### 2.1. Transgenic Rice Lines Enhanced Resistance to Rice Blast

**Figure 1:**
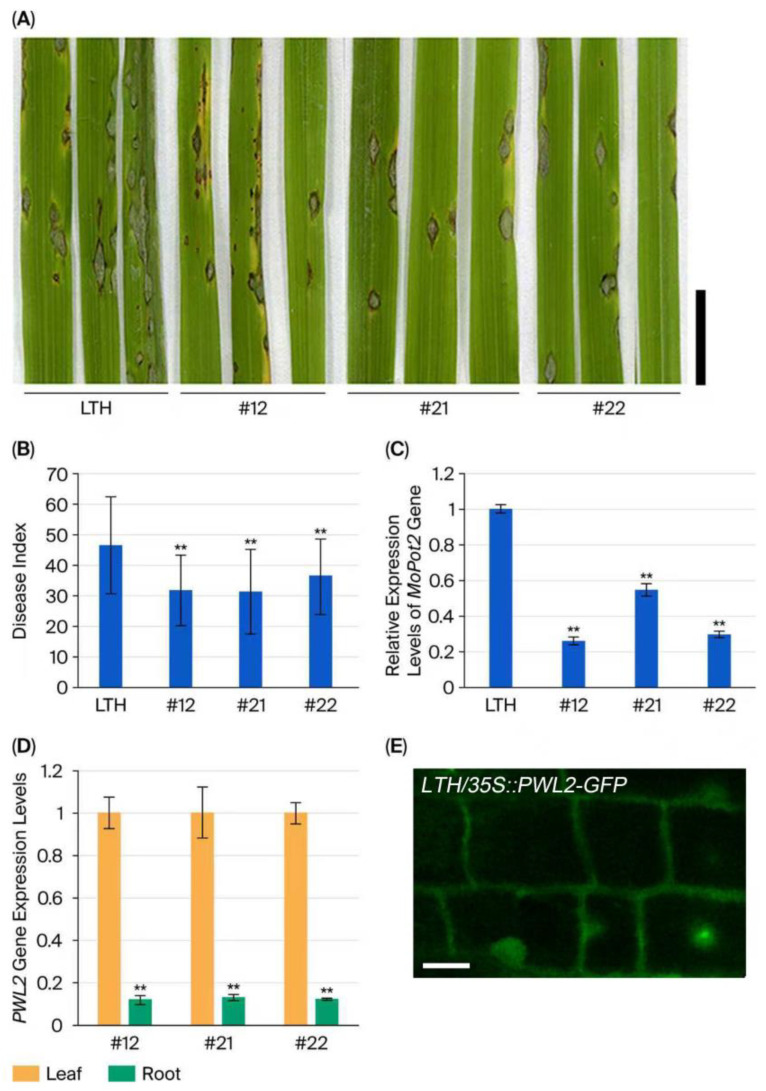
Rice lines overexpressing *PWL2* induced rice resistance to *M. oryzae* infection. Rice seedlings were inoculated with *M. oryzae* strain GUY11. Disease lesion on the leaves of the rice line LTH and transgenic rice lines #12, #21 and #22 (**A**); the quantification of the disease index in the leaves of wild-type rice LTH and transgenic rice lines #12, #21 and #22 (*n*_LTH_ = 58, *n*_#12_ = 47, *n*_#21_ = 35, and *n*_#22_ = 38) (**B**); The expression levels of the *MoPot2* gene in the leaves of wild-type and transgenic rice lines #12, #21 and #22, determined by real-time PCR (**C**); the expression profiles of the *PWL2* gene in the leaves and roots of transgenic rice lines #12, #21 and #22 were determined by real-time PCR (**D**); The *Actin7* gene was used as an internal control. Subcellular localization of PWL2-GFP fusion protein in transgenic rice lines (**E**). Data are means ± SD. ** *p* < 0.01 (*t*-test). Scale bars = 1 cm (in image (**A**)) or 10 μm (in image (**E**)).

To further detect the function of PWL2 in the rice response to *M. oryzae* infection, three rice lines overexpressing the *PWL2-GFP* fusion gene, namely #12 #21 and #22, were randomly selected and inoculated with *M. oryzae* strain GUY11. Compared with the control rice line LTH, which is sensitive to *M. oryzae* (Figure 1A), transgenic rice lines #12, #21, and #22 exhibited smaller lesions (Figure 1A) accompanied by a lower disease index (Figure 1B). The expression levels of the *MoPot2* gene of *M. oryzae* GUY11 were obviously down-regulated in the leaves of the transgenic rice lines (Figure 1C). In the transgenic rice lines, the *PWL2* gene showed higher expression levels in the leaves than in the roots (Figure 1D). Additionally, it was revealed that the PWL2-GFP fusion protein was localized on the plasma membrane (PM) and cytoplasm (Figure 1E), as observed in previous reports [11,31].

### 2.2. SA Treatment Induced Rice Resistance to Blast Disease

**Figure 2:**
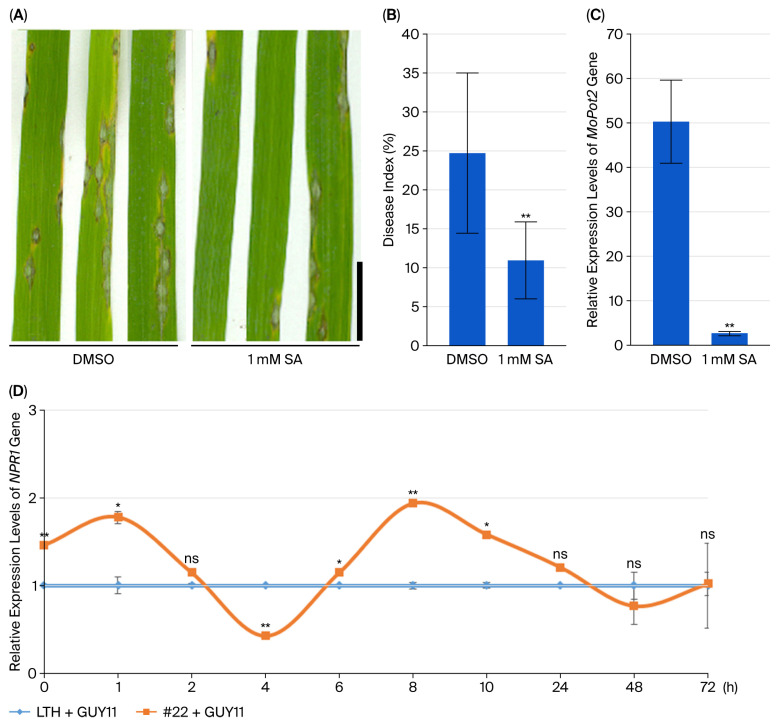
SA treatment induces rice defense against rice blast. Rice LTH was inoculated with *M. oryzae* strain GUY11 and treated with 1 mM SA and DMSO (**A**); Quantification of the disease index in rice LTH treated with 1 mM SA or DMSO (*n*_DMSO_ = 17, *n*_SA_ = 20) (**B**); The expression levels of the *MoPot2* (**C**) and *NPR1* (**D**) genes in the leaves of wild-type rice LTH and *PWL2*-overexpressing transgenic rice lines #22 inoculated with *M. oryzae* strain GUY11 were checked by real-time PCR. The *Actin7* gene was used as an internal control. Data are means ± SD. * *p* < 0.05, ** *p* < 0.01 (Student’s *t*-test for disease index analysis, SPSS analysis for differential gene expression). ns = no significance, h = hour, GUY11 = rice seedlings were inoculated with *M. oryzae* strain GUY11, scale bar = 1 cm.

To detect the role of SA in rice resistance against blast disease, we treated rice plants with exogenous SA and observed their disease resistance. Compared with the control treated with DMSO, rice LTH inoculated with the fungus GUY11 and treated with SA showed smaller lesions (Figure 2A), a lower disease index (Figure 2B) and a significantly down-regulated level of the *MoPot2* gene of *M. oryzae* GUY11 in rice leaves (Figure 2C). To check whether rice lines overexpressing the *PWL2* gene inoculated with the fungus GUY11 triggered SA-mediated immune response, the level of the *NPR1* gene in transgenic rice lines was examined. Compared to rice LTH inoculated with fungus GUY11, the expression level of *NPR1* in *PWL2*-overexpressing rice line #22 increased after inoculation with fungus GUY11, peaked at 1 h post-inoculation (hpi), and subsequently declined. At 2 hpi, *NPR1* expression showed no significant difference compared to the control. It reached the lowest level at 4 hpi and then increased, became significantly higher than the control at 6 hpi, and peaked again at 8 hpi. Expression then decreased at 10 hpi, although it remained significantly higher than in the control and continued to decline thereafter. The *NPR1* gene expression showed no significant difference compared to the control at 24, 48, or 72 hpi (Figure 2D). It showed that SA signaling was activated within 24 hpi in the early stage of *M. oryzae* infection.

### 2.3. PWL2-Derived siRNA Was Detected in the PWL2-Overexpressing Transgenic Rice Lines Inoculated with Fungus GUY11

**Figure 3:**
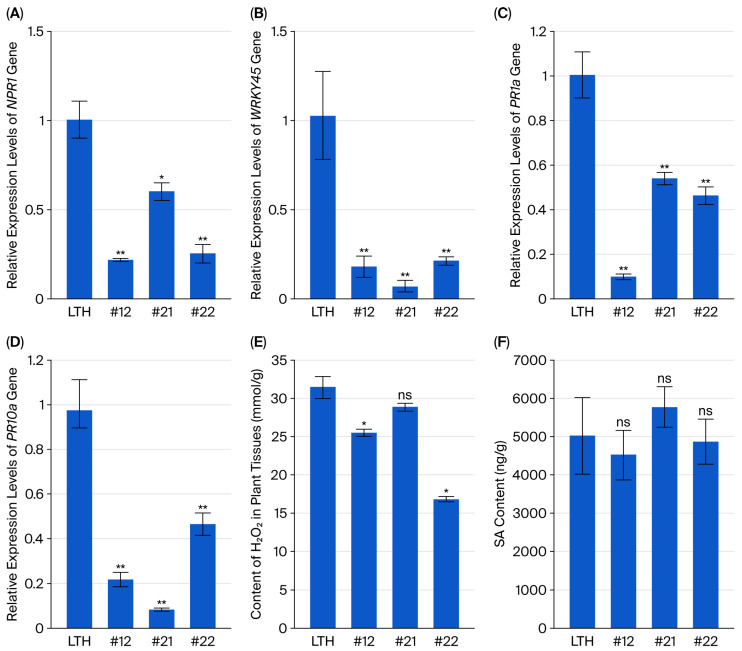
The expression levels of SA signaling-related genes, the H_2_O_2_ and SA contents in the transgenic rice lines. The expression levels of the *NPR1* (**A**), *WRKY45* (**B**), *PR1a* (**C**) and *PR10a* (**D**) genes in the leaves of wild-type rice LTH and transgenic lines, #12, #21, and #22 inoculated with GUY11 were analyzed using real-time PCR. The *Actin7* gene was used as an internal control. The contents of H_2_O_2_ (**E**) and SA (**F**) in the leaves of transgenic lines LTH, #12, #21 and #22 inoculated with GUY11 were quantified. Data are means ± SD. * *p* < 0.05, ** *p* < 0.01, ns = no significance.

To further detect the mechanisms of rice blast resistance, we checked gene expression in rice during the late stage of infection by *M. oryzae* GUY11. Subsequent analysis revealed that, at 7 dpi, compared with rice LTH infected with the fungus GUY11, the expression levels of *NPR1* (Figure 3A), *WRKY45* (Figure 3B), the pathogenesis-related (PR) genes *PR1a* (Figure 3C) and *PR10* (Figure 3D) and the accumulation of H_2_O_2_ (Figure 3E) were decreased, the SA contents did not change in transgenic lines #12, #21 and #22 infected with the fungus GUY11. These results show that SA signaling and the reactive oxygen species (ROS) pathway were suppressed in the late stage of *M. oryzae* infection.

**Figure 4:**
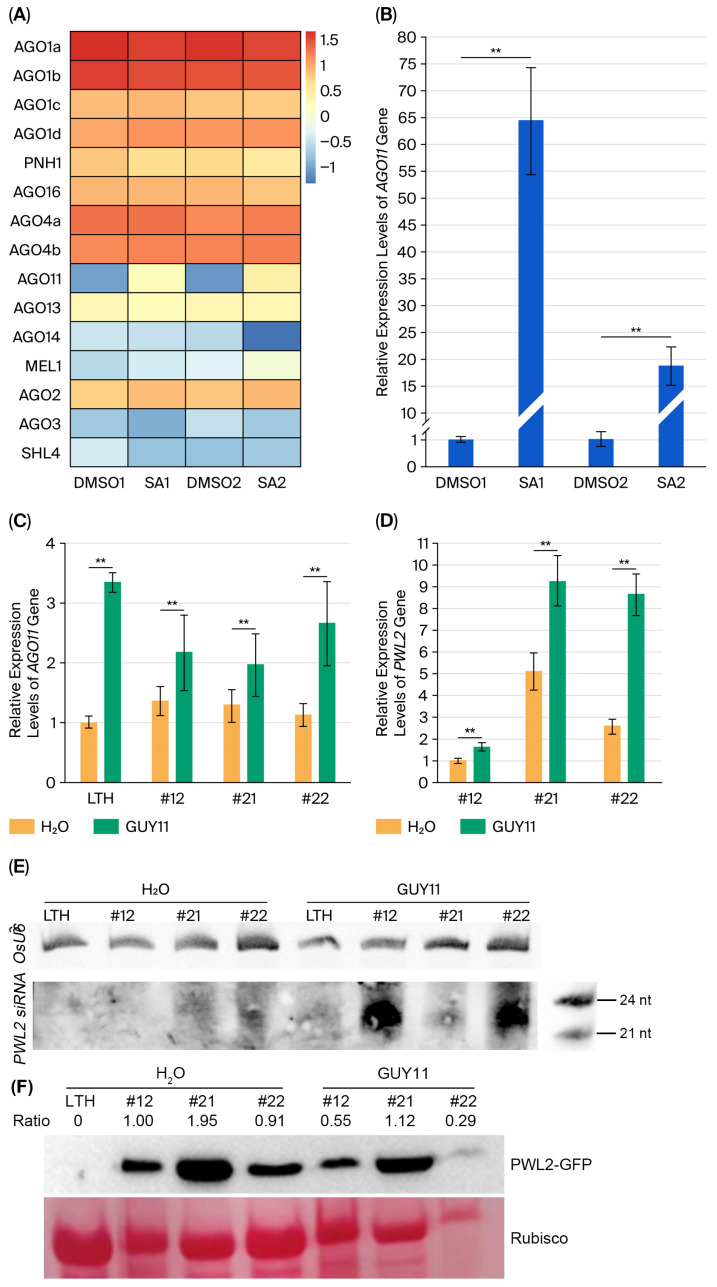
The expression levels of *AGO11*, *PWL2* and *PWL2*-derived siRNA in transgenic lines. Heat maps show the gene expression levels in the roots of rice LTH treated with 2 mM SA (**A**). The relative expression intensity is shown by color-coded: blue, low; yellow, medium; and red, high. The relative expression of the *AGO11* gene in rice root (**B**) and the expression levels of *AGO11* (**C**) and *PWL2* (**D**) in leaves were determined by real-time PCR. *PWL2*-derived siRNA (**E**) was detected by Northern blot in the rice LTH and transgenic lines #12, #21, and #22 inoculated with or without the fungus GUY11. The *Actin7* gene was used as an internal control to detect the *AGO11* and *PWL2* genes, and *OsU6* was used as an internal control to detect the *PWL2*-derived siRNA. Each data was repeated at least three biological replicates. Immunodetection of PWL2-GFP abundance in the indicated samples (**F**). Ponceau staining of Rubisco served as a loading control. The ratio represents the PWL2-GFP band intensity normalized to the Rubisco band intensity measured with Image J (version 1.42q) software. Experiments were independently repeated two times with similar results. Data are means ± SD. ** *p* < 0.01 (SPSS analysis), DMSO: rice seedling treated with DMSO used as a control; DMSO1 and 2: two samples treated with DMSO; SA: rice seedling treated with 2 mM of SA; SA1 and 2: two samples treated with SA; H_2_O: rice seedlings sprayed with sterilized water; GUY11: rice seedlings inoculated with fungus GUY11. FPKM = Fragments Per Kilobase of exon model per Million mapped fragments.

The fungus *M. oryzae* can infect rice roots [32]. To further detect the role of SA in *PWL2*-overexpressing transgenic rice lines against rice blast in the late stage of *M. oryzae* infection, we first analyzed transcriptome data from the roots of rice LTH treated with SA (Figure 4A). We found that *AGO11* expression was significantly upregulated in the roots of SA-treated rice (Figure 4A) and confirmed this using the PCR method (Figure 4B). When we examined the *AGO11* expression in the *PWL2*-overexpressing plants inoculated with fungus GUY11, it showed a substantial increase in the *AGO11* transcript levels in inoculated leaves compared to non-inoculated controls (Figure 4C). Whereas, compared with the control inoculated without the fungus GUY11, the levels of *PWL2* (Figure 4D) in transgenic lines #12, #21 and #22 infected with the fungus GUY11 were increased. We further checked small RNA production in transgenic lines #12, #21 and #22. The expression of *PWL2*-derived small RNA was examined by Northern blot. It showed that a 24 nt *PWL2*-derived siRNA was detected in the leaves of GUY11-inoculated *PWL2*-overexpressing transgenic lines #12, #21 and #22 (Figure 4E). The level of *PWL2* transcripts was increased along with the accumulation of *PWL2*-derived siRNA upon pathogen inoculation (Figure 4D,E). To elucidate the role of *PWL2*-derived siRNA in the translational repression of PWL2 transcripts, we analyzed the abundance of PWL2-GFP protein in transgenic lines before and after *M. oryzae* infection. The results shown that the levels of PWL2-GFP were repressed (Figure 4F) in the late stage of rice resistance.

## 3. Discussion

Effectors are important in the interaction between plants and pathogens. The role of *M. oryzae* effector PWL2 in the rice resistance to blast disease remains partially unclear. In this study, transgenic rice lines overexpressing *PWL2* showed resistance to rice blast. The SA signaling pathway was involved in rice resistance during the early stages of *M. oryzae* infection, and SA could induce the expression of *AGO11*. *PWL2*-derived siRNAs were detected during the later stages of infection, along with increased *AGO11* and *PWL2* levels but decreased PWL2-GFP fusion protein. This implies that there is a temporal transition from SA-dependent defenses in the early infection stage to siRNA-mediated defense dominance during late-stage infection by *M. oryzae*.

SiRNA is involved in plant defense. While PWL2 functions as a virulence factor suppressing immunity in barley [12], its heterologous expression in rice enhances resistance (Figure 1A–C). This indicates that barley and rice may employ distinct mechanisms for PWL2 recognition due to their differing genetic backgrounds. Unlike artificial HIGS strategies targeting essential fungal genes [21,25], these transgenic rice lines produced natural biogenesis of siRNAs derived from *PWL2* (Figure 4E) and increased the level of *PWL2* transcripts (Figure 4D) but decreased PWL2-GFP fusion protein level (Figure 4F), these results suggest that *PWL2*-derived siRNA mediates translational suppression (Figure 4F) rather than transcript degradation in the late-stage rice resistance. Previous studies show that translational repression by siRNAs involves AGO1 and AGO10 [33], with 22 nt small RNAs playing a key role in this process [34]. This study shows that *M. oryzae* infection induces *AGO11* expression, and the 24 nt small RNAs derived from *PWL2*—likely processed by AGO11—may also play a significant role in suppressing protein translation.

AGO proteins are essential for siRNA function [26,27], but the role of AGO11 in plant defense remains unclear. SA cooperates with siRNA-mediated defense in plant antiviral immunity [29,30] and antibacterial defense [35]. SA could induce *AGO11* expression (Figure 4A,B). This finding reveals that SA not only mediates canonical transcriptional defense but also directly participates in RNAi-mediated immunity against fungal effectors. Additionally, H_2_O_2_ accumulation in transgenic rice lines was reduced in the late stage of *M. oryzae* infection (Figure 3E). We also noticed that the cytoplasmic localization of PWL2-GFP (Figure 1E) supports potential recognition by cytoplasmic immune sensors, such as nucleotide-binding leucine-rich repeat (NLR) [36,37]. Furthermore, the endogenous sakuranetin can inhibit the endocytosis of PWL2-GFP, which is localized on the PM (Figure 1E), to enhance resistance when the fungus GUY11 infects rice [11]. Thus, the interplay and underlying mechanisms among the SA signaling pathway, small RNA, ROS, cytoplasmic immune receptors and endocytic pathways that confer blast resistance in transgenic rice lines overexpressing *PWL2* need to be further studied. This study reveals that the temporal decoupling of SA-dependent and RNAi-mediated defenses promotes rice resistance to blast disease and provides a framework for re-evaluating the interaction between phytohormones, including SA, and small RNA in plant defense.

## 4. Materials and Methods 

### 4.1. Plasmid Construction and Rice Transformation and Screening

The *PWL2* gene was PCR-amplified using the primer pair PWL2-FP and PWL2-RP (Table S1) from an expression vector *pBV377* carrying the *PWL2* insert [10]. PCR was performed under denaturation at 94 °C for 2 min, followed by 28 cycles of 98 °C for 10 s, 55 °C for 30 s, 68 °C for 30 s, and a final extension at 68 °C for 5 min. The amplification product was cloned into the vector *pEXT06/g*, placed under the control of the CaMV *35S* promoter, and fused in frame with *EGFP* for expression analysis. The expression vector was constructed at Biogle Company (Hangzhou Biogle Co., Ltd., Changzhou, China). The construct containing the *PWL2* gene fused with the *GFP* gene was named *pEXT06-PWL2::EGFP*. To obtain transgenic LTH (Li Jiang Xin Tuan Hei Gu) rice lines containing the *PWL2-GFP* fusion gene, the construct *pEXT06-PWL2::EGFP* was transformed into *Agrobacterial tumefaciens* strain EHA105. Rice transformation was performed as previously reported [38] at Biogle Company (Hangzhou Biogle Co., Ltd., Changzhou, China) using the *Agrobacterium*-mediated method.

### 4.2. RNA Isolation, cDNA Biosynthesis and RNA Sequencing

The total RNA of leaves and roots of transgenic rice lines was isolated with the EasyPure^®^ Plant RNA Kit (TransGen Biotech, Beijing, China). To synthesize first-strand cDNA, 100 ng each of DNase I-treated RNA, oligo-dT primer and TransScript^®^ II One-Step gDNA Removal and cDNA Synthesis Super Mix (TransGen Biotech) were used to perform the reverse transcription reactions. To investigate the effect of SA on the rice line LTH transcriptome, six-day-old rice LTH seedlings were grown on 1/2 MS medium supplemented with 2 mM SA for 4 days, with DMSO-treated plants serving as controls. Root tissues were then collected for transcriptome sequencing, and the data was deposited in the Genome Sequence Archive (http://gsa.big.ac.cn, accessed on 1 December 2021) under accession number CRA002673 [39]. The FPKM (Fragments Per Kilobase of exon model per Million mapped fragments) value was used to show gene expression level.

### 4.3. Real-Time PCR Analysis

The relative quantitative expression levels of the *PWL2*, *NPR1*, *AGO11*, *PR1a*, *PR10a*, *WRKY45* and *MoPot2* [40] genes were determined using an ABI QuantStudio 7 Flex Real-Time PCR System (Applied Biosystems, Foster City, CA, USA). The 10 μL reaction mixture was prepared with PowerUp^TM^ SYBR^TM^ Green Master Mix (Thermo Fisher Scientific, Waltham, MA, USA) containing the gene-specific primer pair (Table S1) for the *PWL2*, *NPR1*, *AGO11*, *PR1a*, *PR10a* and *WRKY45* genes and a cDNA template. We used the primer pair MoPot2-rFP/MoPot2-rRP (Table S1) and genomic DNA isolated from rice leaves as templates to check the expression level of the *MoPot2* gene. *Actin7* was generated as the internal control [11] and amplified with the primer pair OsActin-FP and OsActin-RP (Table S1). PCR was performed under denaturation at 95 °C for 2 min, followed by 40 cycles of 95 °C for 45 s, 56–60 °C for 30 s and 72 °C for 1 min. Three biological replicates were repeated. The relative expression level was calculated using the 2^−ΔΔCt^ method. SPSS (Version 19.0, IBM, Inc., Armonk, NY, USA) was used to analyze gene expression differences. *p* < 0.05 indicated a significant difference, and *p* < 0.01 indicated an extremely significant difference.

### 4.4. Subcellular Localization Assay

Transgenic rice lines expressing the *PWL2-GFP* fusion gene were grown on MS medium for 5 days. The *PWL2-GFP* fusion protein in the rice root epidermal cells was observed with a Leica SP5 confocal microscope (Leica SP5; Leica Microsystems, Wetzlar, Germany). Confocal images were obtained with a ×40 objective, the fluorescence of PWL2-GFP was excited at 488 nm, and emission was detected between 640 nm and 691 nm, as described in a previous report [11].

### 4.5. M. oryzae Inoculated Rice Seedlings

Seedlings of wild-type LTH and transgenic rice lines expressing the *PWL2-GFP* fusion gene were grown in a greenhouse at 28 °C. Seedlings with three true leaves were inoculated with a spore suspension of *M. oryzae* strain GUY11 at 5 × 10^8^ conidia/mL containing 0.02% Tween 20 for 1, 2, 4, 6, 8, 10, 24, 48, and 72 h and 7 days. To detect SA-induced resistance against rice blast, rice seedlings were inoculated with *M. oryzae* strain GUY11 spores (5 × 10^8^ spores/mL) for 4 days and then sprayed with 1 mM SA. Seedlings treated with DMSO acted as the control. The plant disease resistance was observed at 8 dpi (days post-inoculation), and the disease index was calculated based on the lesion size, as described in a previous study [41,42].

### 4.6. Hydrogen Peroxide (H_2_O_2_) and SA Measurement in Rice Leaves

Leaf segments (1 cm sections adjacent to lesions) were collected from rice plants 7 dpi with *M. oryzae* strain GUY11. Leaf samples were ground, and H_2_O_2_ was extracted using the Hydrogen Peroxide Assay Kit (Nanjing Jiancheng Bioengineering Institute, Nanjing, China) according to the manufacturer’s protocol. The concentration of H_2_O_2_ was quantified spectrophotometrically by measuring absorbance at 405 nm using a microplate reader. To measure the SA content, approximately 200 mg of rice leaves of wild-type LTH and transgenic rice lines expressing the *PWL2-GFP* fusion gene were collected in FastPrep tubes containing 0.9 g of FastPrep matrix, flash-frozen in liquid nitrogen, and grinded to a powder. One milliliter of ethyl acetate spiked with 200 ng of D4-SA was used as the internal standard for SA was added and extracted from each sample. Supernatants were evaporated to dryness and resuspended with 70% methanol (*v*/*v*). The supernatants were analyzed by HPLC-MS/MS (LCMS-8040, Shimadzu, Kyoto, Japan), and the SA content was quantified by comparing its peak area with the peak area of its respective internal standard. Five biological replicates were repeated. The SA concentration was measured by referring to a published report [43].

### 4.7. Small RNA Gel Blot

Small RNA gel blot was performed as previously described [44]. Total RNAs were extracted using Trizol reagent (Invitrogen, Carlsbad, CA, USA) from rice leaves. To analyze small RNAs, 20 μg of the total RNAs was separated on a 15% polyacrylamide/7 M urea denaturing gel and subsequently transferred to a nylon membrane (Cat. no. 11417240001, Roche, Basel, Switzerland). RNA was cross-linked to the membrane using UV irradiation. The membrane was then hybridized with biotin-labeled probes that were complementary to target small RNAs in the PerfectHybTM Plus hybridization buffer (Cat. no. H7033, Sigma-Aldrich, St. Louis, MI,, USA) at 40 °C overnight. After several washes to remove excess probes, the signals of immobilized nucleic acids were detected using the Nucleic Acid Detection Module Kit (Cat. no. 89880, Thermo Fisher, Waltham, MA, USA). The abundance of *OsU6* was used as an internal control to normalize the relative accumulation levels of target small RNAs. To obtain probes for the *PWL2* gene, we used biotin-labeled primers (PWL2-BIO-F: 5′-Bio-ATCCTCCCTTTTGCTTTGG-3′, PWL2-BIO-R: 5′-Bio-TCTTCTCGCTGTTCACGGT-3′) to amplify the target sequence via PCR, followed by high-temperature denaturation to generate single-stranded, biotin-labeled *PWL2* probes. Additionally, a biotin-labeled probe for the *OsU6* gene (5′-Bio-TGTATCGTTCCAATTTTATCGGATGT-3′) was obtained by artificial synthesis.

### 4.8. Protein Extraction and Immunoblotting

To extract total proteins, fresh rice leaves were ground into fine powder in liquid nitrogen and homogenized in 2 × SDS loading buffer (100 mM Tris-HCl, pH 6.8, 20% [*v*/*v*] glycerol, 4% [*w*/*v*] SDS, 0.02% [*w*/*v*] Bromophenol Blue, and 100 mM DTT). The extracts were boiled for 10 min and then centrifuged at 12,000 rpm for 10 min at 4 °C to remove debris. The cleared supernatants were collected and resolved on an 8% SDS-PAGE gel by electrophoresis and transferred to PVDF membranes at 180 mA for 2 h using Trans–Blot apparatus (Cat. no. VE186, Tanon, Shanghai, China) at 4 °C. Signals were visualized with super sensitive ECL luminescence regent (Cat. no. MA0186-1, MeilunBio, Dalian, China), and images were captured using a Tanon 4600 SF Chemiluminescent imaging system (Tanon, Shanghai, China). Antibodies against the PWL2-GFP fusion protein were used for Western blot: anti-GFP antibody (Cat. no. AE011, Abclonal, Wuhan, China; 1:2500 dilution) and Goat anti-Rabbit IgG (H + L) secondary antibody, HRP (Cat. no. 31460, Thermo, Waltham, MA, USA; 1:10,000 dilution). Quantification of protein signal was performed using Image J (Version 1.42q) software.

### 4.9. Accession Numbers

Sequence data for the *PWL2*, *MoPot2*, *Actin7*, *NPR1*, *WRKY45*, *PR1a*, *PR10a*, *AGO1a*, *AGO1b*, *AGO1c*, *AGO1d*, *AGO2*, *AGO3*, *AGO4a*, *AGO4b*, *AGO11*, *AGO13*, *AGO14*, *AGO16*, *MEL1*, *PNH1* and *SHL4* genes described in this study can be found in the NCBI database under the following accession numbers: U26313.1, Z33638.1, Os11g0163100, Os01g09800, Os05g25770, Os07g03710, Os12g36880, Os02g45070, Os04g47870, Os02g58490, Os06g51310, Os04g52540, Os04g52550, Os01g16870, Os04g06770, Os03g47830, Os03g57560, Os07g09020, Os07g16224, Os03g58600, Os06g39640, Os03g33650, respectively.

## Data Availability

The data presented in this study are openly available in the Genome Sequence Archive (GSA) public database (http://gsa.big.ac.cn; accession no. CRA002673, accessed on 1 December 2021).

## Supplementary Materials

The following supporting information can be downloaded at: https://www.mdpi.com/article/10.3390/plants14213312/s1, Table S1: Sequence of gene-specific primers.
