# Supplementary material for: Heterologous Overexpression of Magnaporthe oryzae Effector PWL2 Enhances Rice Blast Resistance via SA-Mediated and PWL2-Derived siRNA Defense
Source: Plants (Basel). 2025 Oct 30;14(21):3312. doi: 10.3390/plants14213312 (PMC12608615; doi:10.3390/plants14213312)
Supplement: Supplementary file 1 [file plants-14-03312-s001.zip › plants-3878231-supplementary.pdf]

Table S1. Sequence of gene-specific primers

Gene	Primer	Sequences of gene-specific primers	Note
MoPot2	MoPot2-rFP	5′-ACGACCCGTCTTTACTTATTTGG-3′	Real-time PCR
	MoPot2-rRP	5′-AAGTAGCGTTGGTTTTGTTGGAT-3′	
Actin-7	OsActin-FP	5′-GAGTATGATGAGTCGGGTCCAG-3′	Real-time PCR
	OsActin-RP	5′-ACACCAACAATCCCAAACAGAG-3′	
PR1a	PR1a-rFP	5′-TGTCGGAGAAGCAGTGGTA-3′	Real-time PCR
	PR1a-rRP	5′-TGATGAAGACGCCGAGGTC-3′	
PR10a	PR10a-rFP	5′- AAGCTCAAGTCACACTCGAC-3′	Real-time PCR
	PR10a-rRP	5′- TCGATCATCTTGAGCATGCC-3′	
WRKY45	WRKY45-rFP	5′- CGGGTAAAACGATCGAAAGA-3′	Real-time PCR
	WRKY45-rRP	5′-GACCCCCAGCTCATAATCAA-3′	
PWL2	PWL2-rFP	5′- AAGGGCTCGGAAGGCGATTT-3′	Real-time PCR
	PWL2-rRP	5′- TATTCGGCGGGTCCATCACC-3′	
PWL2	PWL2-FP	5′-TCAGCAGTCGAAGAGCATGAAATGCAACAACATCAT-3′	Gene clone
	PWL2-RP	5′- TTAGCGTGTGAAGAGCCATAATATTGCAGCCCTCT-3′	
NPR1	NPR1-rFP	5′-GTGCTCGACTACCTCTACAG-3′	Real-time PCR
	NPR1-rRP	5′-AAGTAGCGTTGGTTTTGTTGGAT-3′	

AGO11	AGO11-rFP	5′-TTCCTCCGGTGTACTATGCG-3′	Real-time PCR
	AGO11-rRP	5′-AAGCAGAAGACGATCCGTGG-3′	
